# A case report on late presentation of occult dural lesions 

**DOI:** 10.22088/cjim.8.2.123

**Published:** 2017

**Authors:** Timothy J Cochrane, Gurpreet Singh Ranger

**Affiliations:** 1 Whipps Cross Hospital, London, England; 2 Department of Surgery Dalhousie University, Nova Scotia, Canada.

**Keywords:** Occult dural injury, Meningitis, Cerebrospinal fluid rhinorrhoea, Pneumocephalus

## Abstract

**Background:**

Occult dural injuries are rare and can occur as a result of major or minor head injury. These injuries usually manifest with cerebrospinal fluid rhinorrhea alone, or with meningitis and cerebral abscess, sometimes many years after the original injury.

**Case presentation:**

We present a case of occult dural injury with endocranial complications which occurred in a 34 year old man, with a history of head injury forty-three years ago. The patient presented with a triad of findings; meningitis, CSF rhinorrhoea and pneumocephalus. He was managed conservatively with intravenous antibiotics and observation and made a full recovery. The presence of acute endocranial symptoms and particularly these three findings in a patient with a previous history of head injury, no matter how long it had been should raise suspicion of the presence of an occult dural injury.

**Conclusion:**

It need to retain a high index of suspicion for occult dural injury in patients who present with endocranial symptoms of unknown origin, especially if there is a previous history of head injury.

Head injury, even if minor, can lead to dural injury (1). These lesions can be hard to diagnose and can remain quiescent for many years, eventually presenting with a cerebrospinal fluid leak, or infectious intracranial complications due to ascending infection from the nasal passages. The presence of these injuries is important, as untreated bacterial meningitis has a high mortality and serious complications (1). We present a case of occult dural injury that manifested 43 years after the patient’s original head injury. 

## Case Presentation

An eighty-four year old man attended our accident and emergency department with a severe headache, runny nose and confusion. Detailed history taking was not initially possible at first, but with the arrival of the patient’s relatives, we were able to elucidate further details of his history. The patient’s illness had started two weeks ago with symptoms of sinusitis. He was diagnosed with an upper respiratory tract infection by his general practitioner, and treated with oral antibiotics. We were informed by the relatives that the patient had been “blowing his nose” with increasing frequency over the last two weeks and had not responded to the treatment given by his general practitioner. His rhinorrhoea got much worse, and he then developed a severe headache with worsening confusion over the 24 hours preceding admission. He had sustained severe craniofacial fractures 43 years ago after a serious fall. 

He had undergone neurosurgery for this, but the precise details of the procedures performed were not clear although he had been left with a large scar on the right side of his forehead and an oculomotor nerve palsy. He had otherwise made an excellent recovery from this operation, and had a normal quality of life in full employment until retirement at the usual age. On examination, the patient was very confused. There have been no signs of recent trauma or head injury. Clinical examination revealed neck stiffness and photophobia. His white blood cell count (WBC) and C-reactive protein (CRP) slightly elevated. His chest radiograph was normal. Laboratory testing of the fluid from his nose confirmed it to be CSF; positive for glucose and beta 2-transferrin. The patient was confused and would not allow a lumbar puncture to be performed. Apart from confusion, system examination did not reveal any signs of focal neurological deficit. 

**Figure 1:**
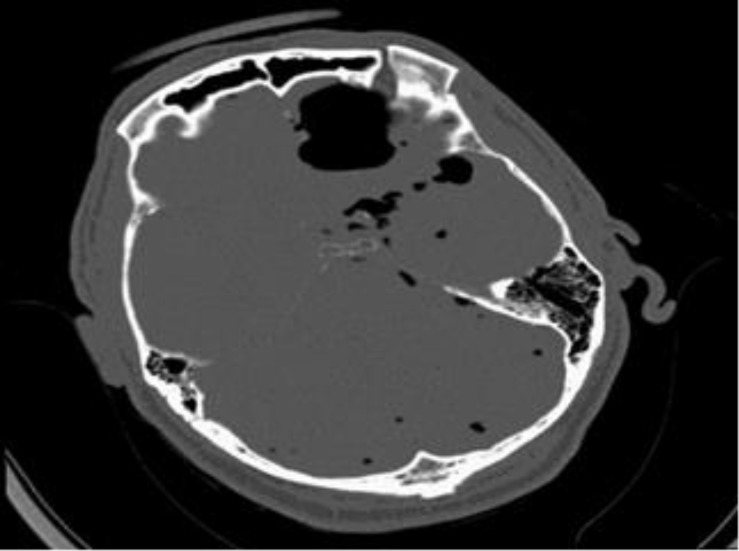
CT of patient on admission demonstrating pneumocranium with old skull fractures

An urgent CT head was performed which showed a pneumocranium and fractures of the frontal bone to the right side of the frontal sinus, involving the superior and medial orbital walls (figure 1). It was initially thought that the patient had an acute head injury, but the absence of physical signs on examination made us question this diagnosis. 

Urgent neurosurgical advice was sought. The skull fractures were considered to be old injuries on review of the scans and a diagnosis of occult dural injury with endocranial complications was made. 

We were advised to treat the patient conservatively with intravenous antibiotics and admission. He recovered rapidly and was discharged after three weeks with advice to avoid straining and maneuvers which could acutely raise intra-abdominal and intrathoracic pressure. He remains well on review 1 year later.

## Discussion

Trauma to the dura can occur from major or minor head injury, although most cases are identified a few days after injury, some of these lesions can go undetected for some time until clinical symptoms supervene. Most often, patients present with recurrent meningitis a spontaneous CSF leak or a combination of the two (1-3). 

It is thought that a head injury of sufficient force can result in a tear of the dural layer. This then leaves a scar or area of potential weakness in the dural layer. Atrophy of the cerebral tissue in this area, for example with age, or injury from repeated microtrauma, for example caused by repeated nose-blowing or sneezing, then allows reopening and establishing a fistula. This then leads to spontaneous efflux of CSF and the ascension of bacteria present within the nasal passages resulting in meningitis. Streptococcus pneumoniae is identified as the responsible pathogen in over 80% of cases (4). Pneumococcal meningitis remains a potential fatal disease, despite the availability of modern antimicrobial therapy and has debilitating sequelae (5).

Schick et al. described their experience of 23 patients who presented with occult dural injuries over a thirteen-year period (1). A traumatic cause was assumed if the patient recalled a history of head injury, or if a skull fracture was detected on imaging or intraoperatively. The mean age of patients was 40 years (range: 12-78) and the interval between the trauma held to be responsible for the occult injury and presentation of the patient with symptoms varied between 1–48 years. Eight patients presented with meningitis, a further eight with CSF rhinorrhoea alone, and a further five with both rhinorrhoea and meningitis. In this series, 13 (56.5%) patients had suffered from more than one episode of bacterial meningitis before occult dural lesion was identified.

There were no patients with triad of pneumocranium, meningitis and CSF leak. Dural defects were repaired operatively in all cases. In a similar paper, Okada et al. reported two cases of CSF rhinorrhoea post trauma, the longest case was 30 years after injury, the other was 10 years (2). Other authors have described comparable experiences (3, 6). Our study differs from Okada et al.’s in which our patient presented 43 years after his initial head injury which is towards the late end of the range described by Shick et al. There was also triad of pneumocephalus, meningitis and CSF leak but not reported in either studies. Previous studies have indicated that computerized tomography is the most reliable means of detecting occult dural lesions but it is accepted that in many cases, the dural injury responsible for presentation may only be discovered during operative exploration (1). The role of surgery remains contentious, but Shick et al. advised operative exploration and repair of dural injuries in all patients who present with suggestive symptoms to avoid further endocranial complications. 

We believe our case is unique as our patient presented an extremely long time after his original head injury, with the triad of a large pneumocranium CSF rhinorrhoea and meningitis. There are some limitations on our report – for example, meningitis had to be diagnosed on clinical grounds in the absence of lumbar puncture. We also assume the most likely mechanism in our case was the establishment of a fistula due to repeated microtrauma of a weakness in the dural area from the previous head injury from many years before. CSF rhinorrhoea with pneumocephalus tend to be seen directly after a traumatic injury and occurrence of both with meningitis so many years after a past head injury has not been reported before (7). Our patient had also been managed conservatively and made an uncomplicated recovery. In conclusion,** c**linicians need to retain a high index of suspicion for occult dural injury in patients who present with endocranial symptoms of unknown origin, especially if there is a previous history of head injury, whether it is major or minor. Prompt diagnosis and treatment are required to prevent serious sequelae.
